# Targeted Delivery of 5-Fluorouracil and Sonidegib via Surface-Modified ZIF-8 MOFs for Effective Basal Cell Carcinoma Therapy

**DOI:** 10.3390/pharmaceutics15112594

**Published:** 2023-11-07

**Authors:** Bharath Singh Padya, Gasper Fernandes, Sumukha Hegde, Sanjay Kulkarni, Abhijeet Pandey, Praful Balavant Deshpande, Sheikh F. Ahmad, Dinesh Upadhya, Srinivas Mutalik

**Affiliations:** 1 Department of Pharmaceutics, Manipal College of Pharmaceutical Sciences, Manipal Academy of Higher Education, Manipal 576104, Karnataka, India; bharathpadya06@gmail.com (B.S.P.); fernandesgasper16@gmail.com (G.F.); sanjay987k@gmail.com (S.K.); abhijeet_pandey87@yahoo.com (A.P.); 2 Department of Pharmaceutics Sciences, Vignan Foundation for Science, Technology and Research, Vadlamudi, Guntur 522213, Andhra Pradesh, India; 3 Centre for Molecular Neurosciences, Kasturba Medical College Manipal, Manipal Academy of Higher Education, Manipal 576104, Karnataka, India; sumukha.hegde7@gmail.com (S.H.); dinesh.upadhya@manipal.edu (D.U.); 4 Respiratory R&D, Teva Pharmaceuticals Ireland, Unit 301, IDA Business Park, X91 WK68 Waterford, Ireland; prafuldeshpande@gmail.com; 5 Department of Pharmacology and Toxicology, College of Pharmacy, King Saud University, Riyadh 11451, Saudi Arabia; fashaikh@ksu.edu.sa

**Keywords:** 5-fluorouracil, sonidegib, topical drug delivery, ZIF-8 MOF, basal cell carcinoma

## Abstract

The therapeutic effectiveness of the most widely used anticancer drug 5-fluorouracil (5-FU) is constrained by its high metabolism, short half-life, and rapid drug resistance after chemotherapy. Although various nanodrug delivery systems have been reported for skin cancer therapy, their retention, penetration and targeting are still a matter of concern. Hence, in the current study, a topical gel formulation that contains a metal-organic framework (zeolitic imidazole framework; ZIF-8) loaded with 5-FU and a surface modified with sonidegib (SDG; acting as a therapeutic agent as well as a targeting ligand) (5-FU@ZIF-8 MOFs) is developed against DMBA-UV-induced BCC skin cancer in rats. The MOFs were prepared using one-pot synthesis followed by post drug loading and SDG conjugation. The optimized MOFs were incorporated into hyaluronic acid-hydroxypropyl methyl cellulose gel and further subjected to characterization. Enhanced skin deposition of the 5-FU@ZIF-8-SDG MOFs was observed using ex vivo skin permeation studies. Confocal laser microscopy studies showed that 5-FU@ZIF-8-SDG MOFs permeated the skin via the transfollicular pathway. The 5-FU@ZIF-8-SDG MOFs showed stronger cell growth inhibition in A431 cells and good biocompatibility with HaCaT cells. Histopathological studies showed that the efficacy of the optimized MOF gels improved as the epithelial cells manifested modest hyperplasia, nuclear pleomorphism, and dyskeratosis. Additionally, immunohistochemistry and protein expression studies demonstrated the improved effectiveness of the 5-FU@ZIF-8-SDG MOFs, which displayed a considerable reduction in the expression of Bcl-2 protein. Overall, the developed MOF gels showed good potential for the targeted delivery of multifunctional MOFs in topical formulations for treating BCC cancer.

## 1. Introduction

Over 75% to 80% of skin cancer diagnoses are BCC, making it the most prevalent. 5-Fluorouracil (5-FU) is an anticancer drug frequently used to treat skin cancer and actinic keratosis [1]. 5-FU inhibits DNA synthesis by inhibiting thymidylate synthase, an enzyme responsible for synthesising pyrimidine bases in genetic material [2]. Topical drug delivery is an effective method of drug administration because it is simple for patients to use, provides a painless route for drug application, and prevents hepatic first-pass metabolism. Several commercial 5-FU topical formulations in creams or gels have shown serious drawbacks, such as skin irritation, inflammation, and intense itching [3]. The hydrophilic nature of 5-FU makes it difficult for topical application with reduced efficacy due to poor permeation through the stratum corneum [4]. Advanced formulations of 5-FU in the form of liposomes, transferosomes, niosomes [5], microsponge [6], and nanogels [7] have been investigated; however, they have limitations in terms of poor tumour retention and specificity, low encapsulation, penetration, and deposition. Therefore, it is essential to develop the drug’s anticancer effectiveness and enhance its targeting efficacy using a novel carrier. Tumor targeting is possible by conjugating or linking the carrier with a specific molecule that can differentiate normal cells from cancer cells. A good targeting molecule is Sonidegib (SDG), which is used for the treatment of BCC by blocking the Hedgehog signaling system and the SMO mutations that are found in BCC pathogenesis [8]. In the present study, Sonidegib was employed in this study as a targeting ligand as well as a therapeutic molecule in the treatment of BCC.

In this work, metal-organic frameworks (MOFs) were selected as the carrier due to their desirable qualities, such as high porosity, extensive surface area, tuneable shape and structure [9]. MOFs are fabricated from metal ions and organic ligands to form a porous cage-like structure. The high drug loading, low cytotoxicity [10], and versatile functionality due to the sigma bond in the organic ligand make MOFs attractive drug delivery hosts. MOFs have widely been explored for the selective separation of gases [11]. Among the MOFs, the zeolitic imidazole framework (ZIF) or the more representative ZIF-8 has emerged as an ideal drug delivery carrier due to its highly porous and nontoxic nature and good thermal and chemical stability [12]. ZIF-8 MOFs have been explored as potential platforms for various biomedical purposes [13], including bioimaging and diagnosis [14], specific tissue and cell targeting [15], high drug encapsulation [16], enhanced confined area drug concentration [17] and the emergence of drug delivery systems [18]. Since ZIF-8 MOFs are porous in structure, it is possible to load fluorescent or small drug molecules and proteins into the core-shell MOFs by adjusting the pore sizes [19] for targeted imaging and delivery. Prior research has shown that hydrophilic or hydrophobic drugs can be loaded into ZIF-8, and drug ligands or linkers can be complexed with the outer surface of the MOFs for combination or multimodal therapy of cancer [20]. To facilitate topical delivery on the skin, the carrier must be mixed with a base such as a cream, gel or ointment.

The use of topical gel formulations in the pharmaceutical field has increased due to their advantageous properties, such as being easily spreadable, greaseless, thixotropic, easily removed, emollient, and water solubility. Hydroxypropyl methylcellulose (HPMC) is a water-soluble cellulose derivative. It is used as a hydrophilic gel matrix for sustained release topical delivery in addition to its potential to control swelling and crosslinking. Furthermore, due to its nontoxicity, ease of compaction, good biocompatibility, and tolerance to high levels of drug loading, HPMC is preferred in drug formulation and delivery. Hyaluronic acid (HA) is a glycosaminoglycan found in endothelial, neural, and connective tissues. It has unique characteristics, such as a highly hydrophilic nature, thixotropy, nontoxicity, and nonimmunogenicity, and it does not degrade to toxic products. These distinct properties are advantageous in wound care dressing materials. HA has also been explored for a variety of biomedical applications, including lubrication and mechanical integrity of arthritic joints, as a surgical aid in ocular surgery, drug carrier agent, and facilitating surgical wound healing. Because of its water-soluble viscous consistency and nonallergenic tissue friendliness, HA has recently been seen in skin-care products such as facial moisturizers. Hence, the dual advantages of HA and HPMC have been explored in this research [21].

Thus, the present research explores a multifunctional drug delivery system for targeted delivery of 5-FU and SDG in the treatment of skin cancer, where SDG acts as a targeting ligand as well as a therapeutic agent. The use of ZIF-8 MOFs loaded with 5-FU followed by functionalization with SDG and incorporation in a gel for skin cancer treatment is reported for the first time in this study. Since ZIF-8 hydrolyses to zinc ions and imidazolate ions [22], zinc ions can promote apoptosis by boosting intracellular reactive oxygen species (ROS) production, which can contribute to the overall treatment of skin cancer [23]. Here, ZIF-8 MOFs were synthesized using a one-pot synthesis by reacting Zn(NO_3_)_2_ with 2-methylimidazole in water. It was observed that the porous nature of the ZIF-8 MOF served as the core for higher 5-FU loading and was further modified by surface complexation with SDG to form 5-FU@ZIF-8-SDG MOFs for targeted drug delivery of the formulation. To enhance topical retention, 5-FU@ZIF-8-SDG MOFs were incorporated into the gel, which was composed of hydroxypropyl methyl cellulose (HPMC; 2%) and hyaluronic acid (HA; 0.5%). The formed nanoparticles were extensively characterized and evaluated both in vitro and in vivo. Moreover, these multifunctional carriers, which may be used for both therapy and targeting, have the potential to overcome the current restrictions on skin cancer treatment.

## 2. Materials and Methods

### 2.1. Materials

2-Methyl imidazole (99%; Cat. No. M50850), zinc nitrate hexahydrate (Zn(NO_3_)_2_.6H_2_O) (98%; Cat. No. 228737), 5-fluorouracil (99.8%; Cat. No. F6627), quercetin, HPMC, propylene glycol (PG), and triethanolamine (TEA) were purchased from Sigma Aldrich, Mumbai, India. HA was obtained as a gift sample from Novozymes, Bagsværd, Denmark. All other chemicals and solvents used were of analytical grade.

### 2.2. Methods

#### 2.2.1. Synthesis and Activation of ZIF-8 MOFs for Post Loading of the Drug

A “one-pot” synthesis method was employed for synthesizing ZIF-8 MOFs with slight modification, as reported by Hoopes et al. [24]. In brief, 2.0 g 2-methylimidazole was added to deionized (DI) water and stirred utilizing a magnetic stirrer to obtain solution I, and 0.2 g Zn (NO_3_)_2_.6H_2_O was added to DI water to obtain solution II. ZIF-8 formation was achieved by the gradual addition of solution II to solution I and was stirred for 15 min at room temperature (RT). Centrifugation (15,000 rpm) was used to extract the synthesized ZIF-8 nanoparticles, which were then washed thrice with DI water. The collected sample was dried for 24 h at 60 °C [25,26]. The activation of the ZIF-8 MOFs was performed by heating ZIF-8 at 160 °C for 24 h, which helps in activating the pores and enhancing the drug loading inside the framework [27]. 5-FU (100 mg) and ZIF-8 MOF (100 mg) were dispersed in methanol (10 mL) separately, followed by dropwise addition of the drug solution to the MOF solution under sonication for 15 min. The final solution was allowed to stir at RT for 2 h, followed by centrifugation to retrieve the drug-loaded MOFs and washing thrice with DI water. The final product was dried at 60 °C for 24 h in a vacuum oven.

#### 2.2.2. Formulation of SDG Complexation with 5-FU@ZIF-8 MOFs and Loading into the Gel

The prepared 5-FU@ZIF-8 MOFs (100 mg) were added to methanol (10 mL) and sonicated for 5 min using an ultrasonic bath sonicator. To the dispersed 5-FU@ZIF-8 MOF solution in methanol, SDG (12 mg) was slowly added under sonication, and the dispersion was further stirred for 6 h to form a complex (5-FU@ZIF-8-SDG). The prepared product was dialyzed against water for 2 h to remove any unreacted drug or targeting agent. The final product was dried at 60 °C for 24 h in a vacuum oven.

#### 2.2.3. Characterization of the ZIF-8 MOFs

The particle size, polydispersity index, and zeta potential of all the ZIF-8 MOFs were analysed using a ZetaSizer Nano Series (Nano ZS 3600, Malvern instruments Pvt. Ltd., Malvern, UK) [28,29]. FT-IR spectra of the samples were analysed by a BRUKER ALPHA II Spectrophotometer (BRUKER, Ettlingen, Germany) in the region of 400 to 4000 cm^−1^ [30]. DSC analysis was performed at 25 to 300 °C using a DSC 60 Plus (Shimadzu, Kyoto, Japan) by sealing samples in an aluminium pan and keeping an empty sealed aluminium pan blank [31]. XRD analysis was performed to study the complexation of drugs with ZIF-8 MOFs using a Miniflex 600 X-ray diffractometer (Ultima IV, Rigaku, Tokyo, Japan) [26]. The shape and surface morphology of the ZIF-8 MOFs were studied utilizing SEM (FEI Quanta 200 ESEM FEG, Isafjordsgatan, Sweden) and TEM (JEM-2100, Tokyo, Japan). AFM analyses were carried out using NT-Integra, Integra, Drive Hudson, NH, USA, at a 4.0 Hz scan rate [32].

#### 2.2.4. Entrapment Efficiency of 5-Fluorouracil and SDG

The entrapment efficiency of 5-FU in the core and SDG on the surface of ZIF-8 MOFs were determined using HPLC as described in our earlier report [33]. 5-FU@ZIF-8 MOFs (10 mg) were placed in an acetate buffer solution at pH 5.0 and stirred at RT for 24 h to break the Zn-imidazole complex. The obtained samples were centrifuged at 10,000 rpm for 30 min, and the supernatant was filtered using a 0.22 µm filter (Corning^®^, New York, NY, USA). The amount of 5-FU and SDG present in the sample was analysed by HPLC after appropriate dilutions. The encapsulation efficiency was calculated using the formula given below:

$$\mathrm{EE}\;(\%)=\frac{Amount\;of\;drug\;obtained}{Theroretical\;amount\;of\;drug\;added}\times 100$$

#### 2.2.5. Preparation of 2% HPMC and 0.5% HA Combination Gel

The HPMC (2%) and HA (0.5%) combination gel was optimized after several trials with different concentrations of HPMC (1%, 1.5%, 2% and 2.5%), as shown in Table 1. Based on the consistency and other gel evaluation parameters, namely, viscosity and spreadability, 2% HPMC and 0.5% HA combination gels were finalized. The gel was prepared by soaking the desired amount of HPMC (200 mg) and HA (50 mg) in warm water (10 mL) overnight at room temperature. Propylene glycol (2% *w*/*v*) was added as a cosolvent along with methylparaben (0.02%) and propylparaben (0.01%) as preservatives. The pH of the gel was adjusted using triethanolamine to 6.4 ± 0.5. The optimized MOFs (800 mg) containing 5-FU (500 mg) and SDG (80 mg) were added to a gel composed of 2% HPMC and 0.5% HA.

#### 2.2.6. Physicochemical Evaluation of the Gels

The gels were inspected manually for clarity, colour, and the presence of any particles. The pH of the gel was determined using a pH meter. The viscosity of the gel was determined by a Brookfield viscometer (LVDV-II+P, Brookfield Engineering Laboratories, Middleboro, MA, USA) using an F-96 (T-bar) Spindle. The viscosity was measured at room temperature at rpm values such as 5, 10, 20 and 100 rpm. The spreadability test was performed by taking for the gel that was placed between glass slides and pressed by putting 200 g weight for 1 min to uniform thickness, and an increase in the area was measured [34]. The drug content in the gel was determined by dispersing the gel formulation (100 mg) in 5 mL of methanol followed by sonication. The sample was then centrifuged for 10 min at 10,000 rpm, and the supernatant solution was appropriately diluted with an aqueous mobile phase. The drug concentration in the gel was determined by HPLC [35].

#### 2.2.7. Ex Vivo Skin Permeation and Retention Studies

The ex vivo skin permeation and retention of 5-FU and SDG from the gel containing plain drugs and 5-FU@ZIF-8-SDG gel formulations were performed using the abdominal skin of Wistar rats [36,37]. The skin sections were kept between the donor and receptor compartments of a vertical diffusion cell. The receptor compartments contained pH 6.8 buffer, and the complete setup was stirred for 1 h at 37 °C to hydrate the skin sections. Drug retention and permeation studies were performed taking 250 mg of gel formulation containing plain drugs dispersed in gel and 5-FU@ZIF-8-SDG in gel (≈12 mg of 5-FU and 5 mg of SDG). The samples were collected at 0.5, 1, 2, 4, 6, 8, 12, and 24 h from the receptor compartment. Each sampling volume was replaced by fresh buffer solutions in the receptor compartment. The concentration of drugs present in the samples was estimated using HPLC [33]. The area of the skin section exposed to diffusion was excised, washed, and homogenized after study completion. The drugs (5-FU and SDG) retained in the skin were extracted in methanol and filtered using a 0.45 µ filter to estimate the drug retained in the skin using HPLC [33].

#### 2.2.8. Depth of Penetration Profiles Using Confocal Laser Scanning Microscopy (CLSM)

The surface of the skin (previously treated with gel of plain drugs and gel containing 5-FU@ZIF-8-SDG for 6 h) was stained with rhodamine B dye to differentiate the epidermis from dermal surfaces and subjected to confocal microscopy. A confocal microscope (SP8, Leica Microsystems, Wetzlar, Germany) equipped with an argon-krypton laser was used to image the skin samples. Scanning was carried out at an excitation wavelength of 488 nm, and an oil immersion objective was used to capture the images. Initially, samples were captured in the x-y plane that was parallel to the surface of the skin, and then imaging with the maximum fluorescence along the x-z axis was identified. The figures from consecutive x-y portions were applied to construct an x-z planar cross-section. The individual image was selected from a set of up to 120 duplicates.

#### 2.2.9. Cytotoxicity Study

The synthesized ZIF-8 MOFs, along with pure drugs and drug-loaded ZIF-8 MOF formulations, were assessed for cell toxicity in human keratinocyte cells (HaCaT) and BCC cells (A431) by MTT assay [38]. The cells were cultured in Dulbecco’s modified Eagle’s medium (DMEM) supplemented with 10% FBS, penicillin (100 µg/mL), streptomycin (100 µg/mL), and amphotericin-B (5 µg/mL) and incubated at 37 °C in a humidifier with 5% CO_2_. The cells were seeded at a density of 2 × 10^4^, treated with the samples and incubated for 48 h. After removal of the media, DMSO was added, the absorbance was recorded at 570 nm using a microplate reader, and cell viability was assessed.

#### 2.2.10. Cellular Uptake Studies

HaCaT cells and A431 cells were seeded in two different 6-well plates at a density of 2 × 10^5^ cells per 2 mL and incubated in a CO_2_ incubator overnight at 37 °C for 24 h. The media of plates were aspirated, and the cells were treated with the test compounds conjugated with FITC solution and controls (FITC solution alone) in 2 mL of culture medium and incubated for 6 h. After the treatment period, the medium from the wells was removed and washed with PBS solution, and 250 μL of trypsin-EDTA solution was immediately added to each plate and incubated at 37 °C for 3–4 min. Two mL of culture medium was added to each well, followed by harvesting the cells directly into polystyrene tubes and centrifugation for 5 min at 300× *g* at 25 °C. After decanting the supernatant, the pellet was washed with PBS. The cells were then analysed by flow cytometry utilizing a 488 nm laser for excitation and detection at 535 nm.

#### 2.2.11. Primary Skin Irritation Studies

For the experiment, the animals (n = 6) were divided into the following different groups:

Group 1: Control (No treatment);
Group 2: Positive control (0.8% formaldehyde solution);
Group 3: 5-FU and SDG dispersed 2% HPMC and 0.5% HA combination gel;
Group 4: ZIF-8 MOFs dispersed in 2% HPMC and 0.5% HA combination gel;
Group 5: 5-FU@ZIF-8-SDG complex dispersed 2% HPMC and 0.5% HA combination gel. The fur from the posterior portion (2 × 2 cm^2^) of the animals was removed by an electric trimmer, and the gels were applied on the shaved back portion of the rat skin (n = 3) (≈2 cm^2^) sequentially for 7 days [39]. Throughout the application period, the skin of the animals was examined daily for inflammation, irritation, or redness at the application site. Scoring was performed as per the Draize evaluation of dermal reactions for erythema and edema [40,41]. By utilizing the formula below, the primary irritation index was calculated:

$$\mathrm{Primary}\;\mathrm{Irritation}\;\mathrm{Index}\;(\mathrm{PII})=\frac{Score\;of\;erythema+Score\;for\;edema}{Total\;Score}\times 100$$

The gel-treated skin area was also subjected to histopathological examination using haematoxylin (H) and eosin (E) staining and was observed for inflammation and oedema. The histopathological scoring was performed as follows: slight = +, moderate = ++ and severe = +++ [40,41].

#### 2.2.12. In Vivo Pharmacodynamic Studies

The in vivo experiments were conducted using male Wistar rats with approval from the Institutional Animal Ethics Committee (IAEC) (Reference No: KMC/03/2019) of the Central Animal Research Centre Facility, Kasturba Medical College, Manipal, India. Wistar rats weighing between 200 and 220 g were housed in cages. Animals selected for the experiment were allowed to adjust for 2 weeks to laboratory conditions before initiating the experiment. The housed animals were set aside and fed food and water at 25 °C in a conditioned atmosphere. The following is a list of the groups of rats, each group containing six rats:

Group 1: Negative control (No disease-induced, No drug treatment);
Group 2: Positive control (disease induced no treatment);
Group 3: 5-FU dispersed 2% HPMC and 0.5% HA combination gel;
Group 4: SDG dispersed 2% HPMC and 0.5% HA combination gel;
Group 5: 5-FU and SDG dispersed 2% HPMC and 0.5% HA combination gel;
Group 6: ZIF-8 MOFs dispersed in 2% HPMC and 0.5% HA combination gel;
Group 7: 5-FU@ZIF-8 MOFs dispersed in 2% HPMC and 0.5% HA combination gel;
Group 8: 5-FU@ZIF-8-SDG complex dispersed in 2% HPMC and 0.5% HA combination gel; Group 9: Marketed formulation (5% Efudex).

- Induction of cancer (BCC) in animal model: 7,12-Dimethyl-benzanthracene solution (1% *w*/*v*) was freshly prepared in acetone for the induction of BCC in male Wistar rats. Approximately 200 µL of this freshly prepared 1% DMBA solution was topically added to the shaved area of the rat skin and exposed to UV light at a wavelength of 311 nm for 1 min every other day for a period of 3 months. After 3 months of visualization of BCC induction, the skin was excised, and the processed portion of the skin was fixed in Bouin’s fixative and taken for histopathological examination. Hematoxylin and eosin were used for staining, and the pathological changes observed were viewed under a light microscope. Untreated skin was considered the negative control, and skin treated with 1% DMBA along with UV exposure was considered the positive control. The skin sections were observed for dermal masses composed of lobules and cords of tightly crowded cells that were sustained by a fluctuating fibrovascular stroma to confirm BCC induction. Tumor cells from surrounding nests with central necrosis confirm the solid type of BCC induction [42]. The efficacy of the formulations was assessed by visual observation of the BCC-induced area of the skin, histopathological studies of the skin, immunohistochemistry, and protein expression studies by Western blot analysis.
- Visual observation: Visual observation was carried out on the animals of Group 1, Group 2, Group 8 and Group 9. During the treatment period, the animals were visually observed weekly once up to the third week and on the last date of the treatment (30th day) for the reduction in BCC. The treated areas of the skin were photographed every 2 weeks until the completion of the treatment.
- Histopathological studies: The efficacy of the plain drugs and ZIF-8 MOF gel formulations in comparison with marketed products was investigated in rats using histopathological evaluation. On the 30th day of the treatment period, after visual observation, the animals were sacrificed using thiopentone and cervical dislocation. The treated skin area was excised carefully using surgical scissors, and the excised skin was collected and stored in 10% formalin for two days at room temperature. After 48 h, the tissue was transferred into fresh phosphate buffer for 2 h and then transferred into 50% ethanol solution for 24 h. The skin tissue from 50% ethanolic solution was then transferred into 70%, 90%, and 100% ethanol every 24 h and finally transferred into xylene solution until the tissue became transparent. The tissue was then transferred into xylol solution and embedded in parafilm wax for sectioning. Skin tissue sections were made by using a microtome, and 5 µm sections of tissue were collected on positively charged coated glass slides. The skin section was fixed in Bouin’s fixative and taken for histopathological examination. The slides were stained with hematoxylin and eosin and observed for pathological changes that occurred in the treated group compared to the positive control group under a light microscope.
- Immunohistochemistry: The tissues, which were collected for histopathological studies, were also used for immunohistochemistry to identify the suppression of Bcl-2 gene expression in the treated group by fluorescence microscopy. The detailed procedure is given in Supplementary Information S1.1.
- Protein expression study: Western blot analysis was performed in the collected skin tissue to quantify the amount of Bcl-2 protein expressed in different treatment groups. The steps involved in the protein expression study are shown in Supplementary Information S1.2.

#### 2.2.13. Stability Studies

Stability studies of 5-FU@ZIF-8-SDG MOFs and 5-FU@ZIF-8-SDG MOF gels were carried out at 25 ± 2 °C/60 ± 5% RH for 6 months. The samples were filled in amber-colored vials and sealed with rubber closures followed by crimping with aluminum caps. The 5-FU@ZIF-8-SDG gel samples were collected at time intervals of 1, 3, and 6 months and analysed for appearance, pH, viscosity, spreadability and drug content. Similarly, the 5-FU@ZIF-8-SDG MOFs were analysed for drug content [43].

#### 2.2.14. Statistical Analysis

The data were analysed by GraphPad Prism (v. 8.0.1; GraphPad Software, San Diego, CA, USA). To compare the values with the control group, one-way ANOVA was performed, followed by Dunnet’s test and Tukey’s post hoc test. We considered *p* values less than 0.05 to be statistically significant. For immunohistochemistry, the statistical analysis was performed using ANOVA and a nonparametric test by using EZR or SAS 9.4 software. A *p* value < 0.05 was considered significant.

## 3. Results

### 3.1. Formulation and Characterization of 5-FU@ZIF-8-SDG MOFs

The ‘one-pot’ synthesis method was followed for the fabrication of ZIF-8 MOFs, as it produces MOFs with desirable properties in accordance with the zeta potential and particle size. From previous reports [44,45] and preliminary batches, it was found that a ratio of the ion and linker of 1:10 was optimum to form ZIF-8 MOFs. The size of the ZIF-8 MOFs was found to be 68.97 ± 1.52 nm with a low PDI of 0.11 ± 0.05 and zeta potential of 24.4 ± 2.16 mV [46]. After drug loading, it was observed that the particle size increased (140 ± 4.62 nm); however, the homogeneity and zeta potential remained constant. The 5-FU@ZIF-8 MOF has a positive charge on its surface, which is necessary to bind the SDG molecules that bear a negative charge. Overall, the 5-FU@ZIF-8-SDG MOF particle size increased (172.32± 5.62), while the zeta potential decreased (−15.7 ± 2.05), indicating successful complexation of SDGs on the surface of 5-FU@ZIF-8 MOFs [47].

**Figure 1:**
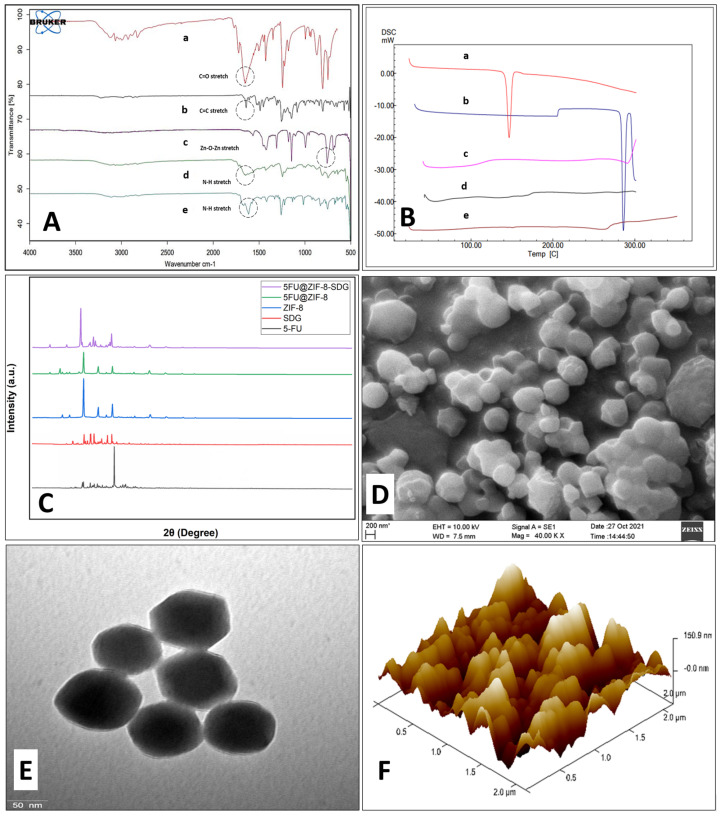
Characterization of MOFs. (**A**) presents the FTIR spectra of different samples: (a) 5−FU, (b) SDG, (c) ZIF−8 MOFs, (d) 5−FU@ZIF-8 and (e) 5−FU@ZIF-8-SDG Complex. (**B**) represents DSC thermograms of different samples: (a) SDG, (b) 5−FU, (c) ZIF−8 MOFs, (d) 5−FU@ZIF-8, and (e) 5−FU@ZIF−8−SDG MOFs. (**C**) represents the XRD pattern of different samples. (**D**) presents an SEM image of ZIF−8 MOFs at the 200 nm scale. (**E**) presents a TEM image of ZIF−8 MOFs at the 50 nm scale and (**F**) represents an AFM image of ZIF−8 MOFs representing the surface roughness. * in figure over “nm” is to denote that “nm” stands for nanometer.

The FTIR spectra of 5-FU, SDG, ZIF-8 MOFs, 5-FU@ZIF-8 and 5-FU@ZIF-8-SDG are shown in Figure 1A. The FTIR spectrum of 5-FU showed stretching vibrations of functional groups C=O at 1650 cm^−1^, −CF at 1242 cm^−1^, and −NH stretching in the 3000–3500 cm^−1^ region [48]. SDG showed mixed stretching vibrations of CH_3_ at 1391–1487 cm^−1^, aromatic C-C at 1383 cm^−1^, and C-H stretch at 1147 cm^−1^ and bending of –OH of the two phenolics and an enolic group at 997 cm^−1^ and 804 cm^−1,^ respectively. The FTIR spectrum of the synthesized ZIF-8 MOFs shows the stretching vibrations of the C–H bonds of the imidazole ring at 2831–3181 cm^−1^. Characteristic in-plane and out-of-plane bending peaks of the imidazole ring were observed at 682–752 and 994–1180 cm^−1^, respectively. The stretching vibrations of Zn–N bonds and C–N bonds were observed at 538 cm^−1^ and 1310–1425 cm^−1,^ respectively [49]. The FTIR spectra of ZIF-8,5-FU@ZIF-8 and 5-FU@ZIF-8-SDG displayed all the major peaks of 5-FU, SDG, and ZIF-8 MOF stretching vibrations, showing successful 5-FU loading and surface complex formation of ZIF-8 with SDG [50]. The peaks at 538 cm^−1^ for Zn–N and 1425 cm^−1^ for the C–N bond are associated with hollow ZIF-8, and the stretching vibrations at 1383–1487 cm^−1^ for -OH are the two phenolic groups correlated with SDG [51]. The N-H stretching vibrations in ZIF-8, 5-FU@ZIF-8 and 5-FU@ZIF-8-SDG are broadened, indicating hydrogen bond formation between the Zn^+^ on the ZIF-8 surface and phenolic OH^−^ groups of SDG by strong electrostatic interactions. This confirms the successful surface complex formation of SDG on the ZIF-8 surface [20].

Figure 1B shows the DSC peaks of SDG, 5-FU, ZIF-8 MOFs, 5-FU@ZIF-8 and 5-FU@ZIF-8-SDG MOFs. The DSC curve of 5-FU showed an intense peak at 286 °C, representing its melting point [52]. No endothermic peak was observed in the drug-loaded 5-FU@ZIF-8 MOFs, indicating that the drug was incorporated into the ZIF-8 MOFs. The melting point of SDG showed an intense peak at 146 °C in the DSC curve, and the same peak was observed in the 5-FU@ZIF-8-SDG MOFs, representing the surface coating of SDG on the 5-FU-loaded ZIF-8 MOFs. DSC data of ZIF-8 MOFs confirmed that there is an absence of peaks.

The crystalline structure of ZIF-8 MOFs was obtained using XRD (Rigaku Ultima IV, Tokyo, Japan). As shown in Figure 1C, the XRD patterns of 5-FU shows intense diffraction peaks at 2.25°, 17.4°, 20.2°, 21.5°, 22.6°, 29.2°, 31.5°, 32.4°, 33.7° and 58.9° in its diffraction pattern [53]. The pure SDG indicated crystalline structure due to peaks observed at 9.9°, 10.7°, 13.4°, 17.6°, 24.2°, 25.0°, 25.9°, and 27.6°. The synthesized hollow ZIF-8 MOFs, showed the characteristic strong peaks with slight change in 2θ values at 12.94°, 14.86°, 18.56°, 22.48°, 24.64°, 26.24°, 32.18°, 36.34° respectively as reported earlier [54]. A slight reduction in the crystallinity of 5FU@ZIF-8 MOFs suggested successful loading of 5-FU into the ZIF-8 MOFs. The crystallinity of 5-FU@ZIF-8-SDG MOFs was not affected by the surface complexation of SDG, which indicated that SDG had minimal effect on the crystallinity of the ZIF-8 MOFs [20].

The surface morphology of ZIF-8 MOFs by SEM analysis (Figure 1D) showed a typical hexagonal shape with well-defined edges and a size distribution with an approximate size of approximately 200 nm [55]. The TEM image shown in Figure 1E indicated the hexagonal shape of the particles [56] along with a uniform size distribution of particles <250 nm, supporting the size range obtained using DLS and SEM. AFM was used to determine the ZIF-8 MOF surface topography and shape, as illustrated in Figure 1F. The particle size was found to be below 250 nm, which is almost in agreement with the size obtained by DLS and TEM. The particles appear aggregated when viewed in a single plane; however, the DLS (PDI) confirmed the homogeneity of the MOFs [57].

The drug entrapped (5-FU) in the core and SDG complexed on the 5-FU@ZIF-8-SDG MOF surface was analysed using HPLC and was found to be 63.0% for 5-FU and 66.6% for SDG. It is plausible to assume that the increased drug loading and complexation are due to the high surface area.

### 3.2. Physicochemical Evaluation of the Gel

Based on the consistency, spreadability, and viscosity of the gel, 2% HPMC and 0.5% HA gel were used as the bases for the preparation of 5-FU@ZIF-8-SDG gel. Different gel formulations, as shown in Table 1, were prepared by dispersing plain drugs 5-FU and SDG or 5-FU@ZIF-8-SDG (equivalent to 5% *w*/*w* of 5-FU) in the gel base. The physicochemical evaluation of the prepared gel formulation was performed, and the results are represented in Table 2.

### 3.3. Ex Vivo Skin Permeation and Retention Studies

**Figure 2:**
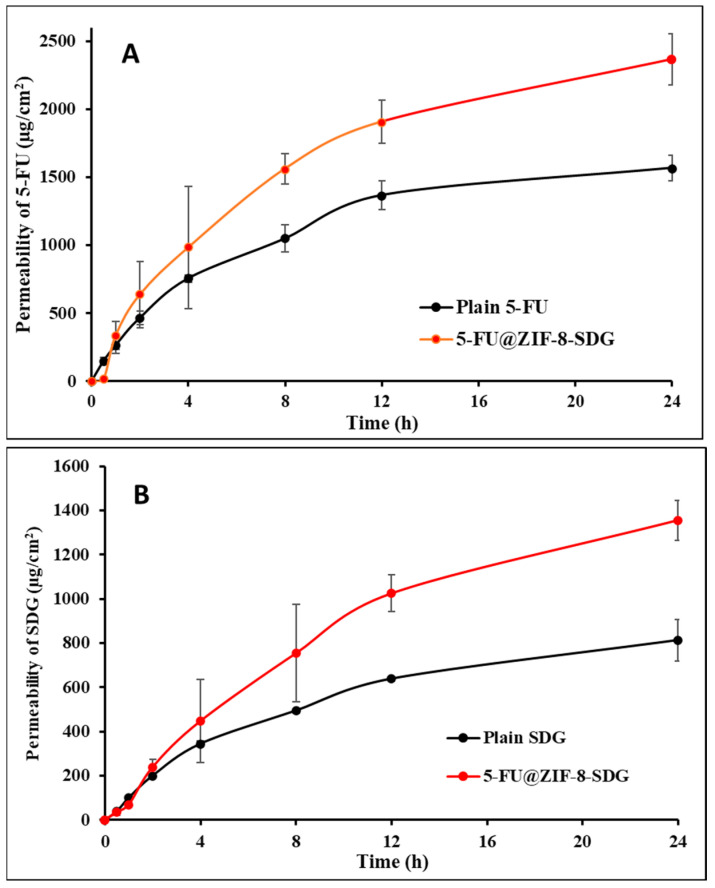
Ex vivo permeation study. (**A**): Permeability of 5-FU from plain drug dispersed gel and 5-FU@ZIF-8-SDG MOF gel formulation at pH 6.8. (**B**): Permeability of SDGs from the plain drug dispersed gel and 5-FU@ZIF-8-SDG MOF gel formulation at pH 6.8. All the points in the graph are presented as Mean ± SD (n = 3).

According to earlier studies, when administered orally, 5-FU and SDG have low bioavailability due to poor membrane permeability and low GIT absorption [58,59]. As a result, topical administration of these drugs would help to achieve the desired pharmacological activity [60]. The primary goal of the permeation study was to evaluate the effect of the 5-FU@ZIF-8-SDG MOF gel on the skin permeation of 5-FU and SDGs. The ex vivo skin permeation of 5-FU and SDG across rat skin of plain drug (5-FU and SDG) dispersed gel formulation and 5-FU@ZIF-8-SDG MOF gel is shown in Figure 2. The permeation parameters (permeability coefficient and drug content in skin) of 5-FU and SDG at pH 6.8 are shown in Table 1. The amount of 5-FU and SDG permeated from the simple gel at pH 6.8 at the end of 24 h (Q_24_) from the pure drug dispersed gel was 1569.22 ± 105.29 µg/cm^2^ (5-FU) and 813.14 ± 294.85 µg/cm^2^ (SDG), while in comparison with the 5-FU@ZIF-8-SDG complex gel, approximately 2896.32 ± 187.50 µg/cm^2^ (5-FU) and 1356.24 ± 90.72 µg/cm^2^ (SDG) permeated at the end of 24 h. Approximately 1.84-fold and 1.66-fold higher permeation was observed for 5-FU and SDG from the optimized formulation at pH 6.8. The Q24 values observed in this study are slightly higher than those reported by our group earlier, which may be due to the use of human epidermis as the membrane in the previous report, which acts as a stronger barrier for drug penetration than the rat skin, which is used in the present study. In comparison to earlier investigations, the permeation of 5-FU through rat abdominal skin was approximately 4-fold higher than that through human epidermal skin because rat skin is 2-10-fold permeable than human skin [61]. As shown in Table 3, the permeability coefficients of 5-FU and SDG observed with the 5-FU@ZIF-8-SDG gel were found to be higher than those of the pure drug-containing gel. Increased skin retention of 5-FU and SDG was associated with an increased permeation rate, and these observations are in accordance with our previous report [40]. The skin retention values of the plain drug dispersed gel formulation at pH 6.8 were 998.16 ± 140.18 µg/cm^2^ (5-FU) and 109.13 ± 10.12 µg/cm^2^ (SDG), while in the 5-FU@ZIF-8-SDG gel, 3898.16 ± 120.18 µg/cm^2^ (5-FU) and 615.26 ± 49.12 µg/cm^2^ (SDG) with 3.9-fold and 5.64-fold increases in skin retention, respectively, were observed. This multifold retention in the skin may be due to multivalent interactions occurring between the MOFs and biological membranes, which may be responsible for higher drug retention in skin [62].

### 3.4. Depth of Penetration by Confocal Laser Scanning Microscopy (CLSM)

**Figure 3:**
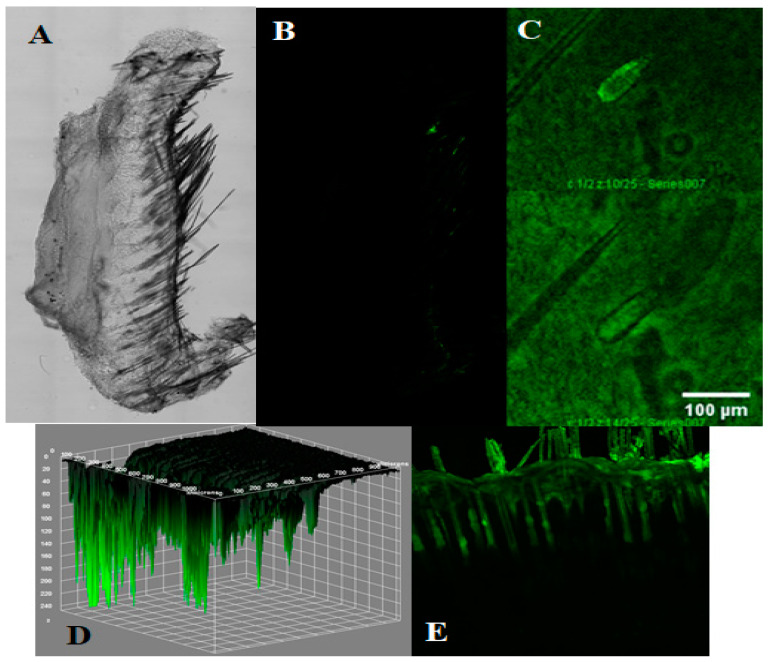
Confocal images of the skin sections obtained at 6 h after treatment with plain FITC or ZIF-8 MOF. (**A**) Control skin, (**B**) plain FITC, (**C**) ZIF-8 MOFs with FITC, (**D**) 3D image indicating the depth of penetration of ZIF-8 MOFs with FITC, (**E**) ZIF-8 MOFs with FITC showing the deposition of MOFs alongside the hair shaft.

The pathway of nanocarrier penetration through the skin is reliant on the physicochemical characteristics of the nanocarrier, of which size is one of the key factors. CLSM was used to visualize the depth of penetration of the optimized MOFs through rat skin. The optical images of the skin treated with plain FITC or MOFs obtained by CLSM are shown in Figure 3. The fluorescence seen with all treatments could be attributable to the fluorescent dye (FITC) and MOFs containing FITC because untreated skin displayed negligible autofluorescence (Figure 3A). Spotty fluorescence was observed with weak intensity to a depth of 200 µm in the skin section treated with plain FITC, as shown in Figure 3B. Skin treated with the gel containing ZIF-8 MOFs loaded with FITC exhibited high intensity of fluorescence up to a depth of approximately 250 µm (Figure 3C). This observation supports high drug retention via MOFs in skin permeation studies. The 3D image (Figure 3D) also shows that the MOFs can permeate up to a depth of approximately 250 µm. Additionally, skin treatment with the gel containing ZIF-8 MOFs loaded with FITC demonstrated a high intensity of fluorescence in the hair follicles and hair shaft, which is indicative of the involvement of the transfollicular route of penetration of the ZIF-8 MOFs. In a previous study, higher fluorescence was observed in hair follicles than in the surrounding tissue when the skin was treated with nanoparticulate gel [12] (Figure 3E). Nanocarriers have been found to utilize two major pathways of transport across skin, viz., the transcellular pathway and the transfollicular (or trans-appendageal) pathway. The intercellular route between corneocytes is a highly tortuous path. This route has been evidenced only for extremely small nanoparticles on the order of a few nm [63]. Moreover, intercorneocyte lipids have been found to impede particle movement along this route. The transfollicular route involving the invasion of particles into hair follicles is thought to be a quicker route into viable skin and is complementary to SC permeation by molecules. Studies on ZIF-8 MOF transport across skin have shown that cationic vesicles primarily utilize the transfollicular route due to the negative charge borne by hair [64]. Therefore, the present study highlights the transfollicular pathway for the penetration of skin by the developed nanocarrier formulations. However, any other factors responsible for the transfollicular permeation of ZIF-8 MOFs may still require detailed study.

### 3.5. Cytotoxicity Study

**Figure 4:**
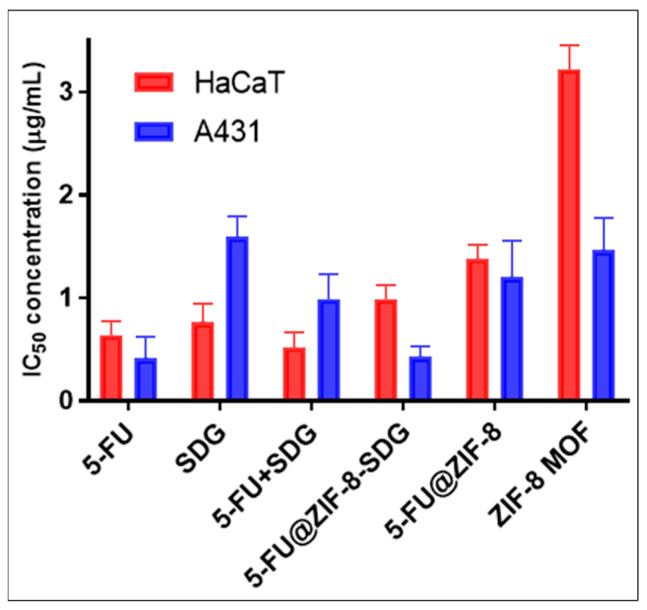
In vitro cytotoxicity assay for plain ZIF-8 MOFs, 5-FU@ZIF-8, and 5-FU@ZIF-8-SDG complex formulations on HaCaT cells and A431 cells.

The cytotoxicity of SDG, 5-FU, 5-FU+SDG, ZIF-8 MOFs, 5-FU@ZIF-8 and 5-FU@ZIF-8-SDG MOFs was evaluated in HaCaT and A431 cells (Figure 4). After incubation for 48 h, the 5-FU@ZIF-8-SDG MOFs showed lower cytotoxicity (based on the IC_50_ values) than the plain drugs or drug combination on HaCaT cells. However, the opposite was observed in A431 cells, wherein 5-FU@ZIF-8-SDG MOFs showed the highest toxicity compared to plain drugs and drug combinations. The ZIF-8 MOFs show negligible cytotoxicity in both cells. Given that HaCaT cells represent normal skin cells and A431 cells represent skin cancer cells, the 5-FU@ZIF-8-SDG MOFs are more toxic to skin cancer cells than to normal cells. Furthermore, we can conclude that 5-FU is slowly released from the MOFs, making it possible to deploy them as effective drug delivery systems against skin cancer.

### 3.6. Cellular Uptake Studies

**Figure 5:**
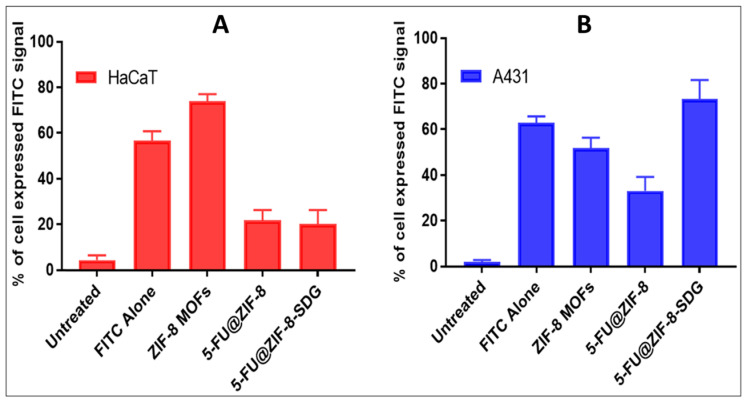
In vitro cell uptake studies. (**A**): In vitro cell uptake studies of ZIF-8 MOFs, 5-FU@ZIF-8, and 5-FU@ZIF-8-SDG complex formulations in HaCaT cells. (**B**): In vitro cell uptake studies of ZIF-8 MOFs, 5-FU@ZIF-8, and 5-FU@ZIF-8-SDG complex formulations in A431 cells.

Cellular uptake studies for plain FITC, ZIF-8 MOFs, 5-FU@ZIF-8 and 5-FU@ZIF-8-SDG MOFs were performed on HaCaT and A431 cells, as shown in Figure 5A,B. The observations strongly suggest that the test compounds (ZIF-8 MOFs, 5-FU@ZIF8 MOFs and 5-FU@ZIF8-SDG MOFs) considerably expressed the FITC signal in HaCaT and A431 cells and confirmed good cellular uptake in both cells. However, when comparing the cellular uptake of the two cells, the 5-FU@ZIF8-SDG MOFs showed higher uptake in the A431 cells due to the presence of SDG on the surface of 5-FU@ZIF8-SDG MOFs, which tends to bind to SMO receptors present on the A431 cells. The absence of SMO receptors on HaCaT cells leads to less cellular uptake compared to A431 cells.

### 3.7. Primary Skin Irritation Studies

Assessment of irritation of pharmaceutical products that are to be applied on the skin is a significant step in the evaluation of their biocompatibility and toxicity. Primary skin irritation studies were performed on Wistar rats for plain 5-FU+SDG gel, ZIF-8 MOF gel and 5-FU@ZIF-8-SDG gel and were compared with the control. With the 5-FU@ZIF-8-SDG gel, no external indications of irritation or inflammation were observed. In comparison to the positive control (formalin 0.8% *v*/*v*), the 5-FU@ZIF-8-SDG MOF gel considerably (*p* > 0.05) reduced the PII ratings for erythema and edema. The results showed that the 5-FU@ZIF-8-SDG gel showed negligible edema and erythema compared to that of formalin (0.8% *v*/*v*). According to the Draize test score, formulations producing scores from 0 to 2 fall under the “nonirritating” to “slightly irritating” category. The 5-FU@ZIF-8 gel and 5-FU@ZIF-8-SDG gels were found to be safe to apply on skin with no or slight irritation, as shown in Table 4.

**Figure 6:**
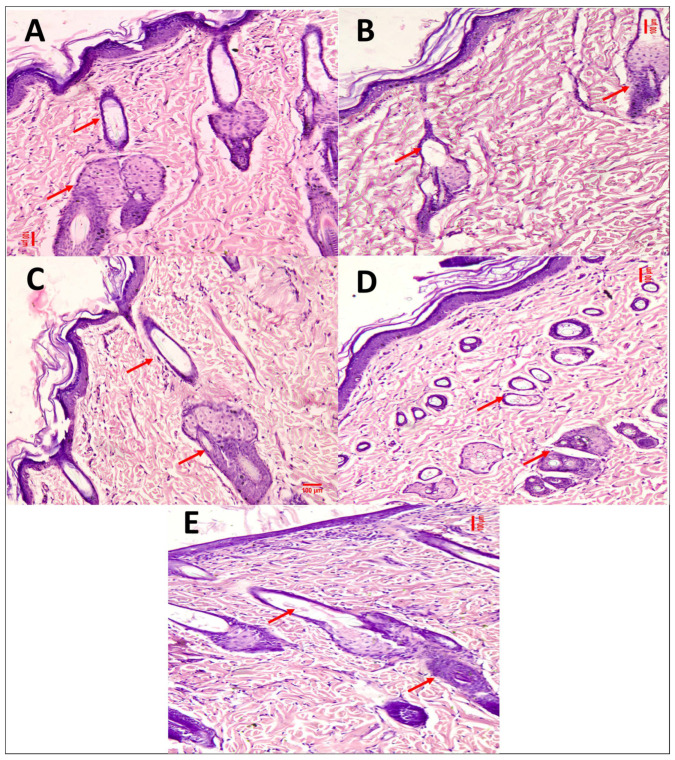
Photomicrographs of histopathological evaluation of rat skin stained with H and E in the primary skin irritation study. (**A**) Positive control, (**B**) Negative control, (**C**) 5-FU@ZIF-8-SDG gel, (**D**) ZIF-8 MOF gel, and (**E**) plain 5-FU and SDG gel.

The histopathological results of the primary skin irritation studies are shown in Table 5 and indicated that normal skin did not show any sign of dermal toxicity. The animals treated with the 5-FU@ZIF-8-SDG complex showed slight degeneration, congestion, necrosis, edema and flammability, indicating no considerable dermal toxic reactions, as shown in Figure 6. Normal epidermis with stratum corneum was found undisturbed in all the skin samples treated with all the gel formulations. The dermis showed a normal pattern of collagen fibre bundles with hair follicles and sebaceous glands. These results showed the greater dermal safety and nontoxic nature of gels.

### 3.8. In Vivo Pharmacodynamics Studies

(a) Visual observation

**Figure 7:**
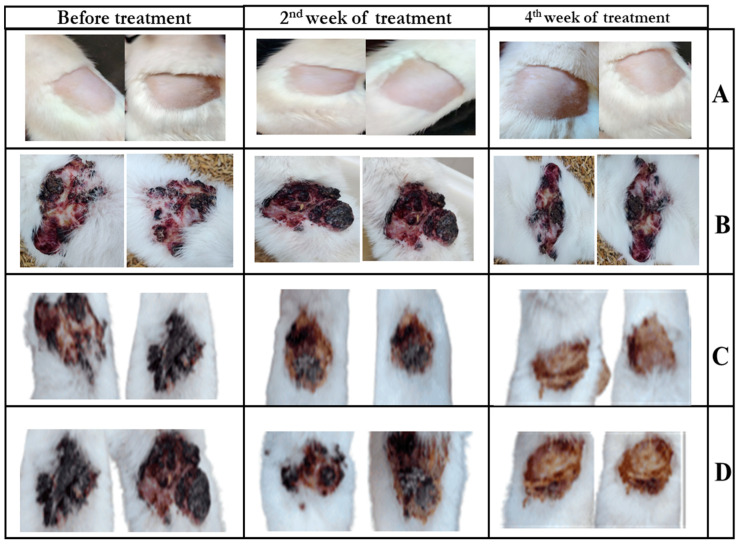
Visual observation of the animals with different treatments. (**A**). Negative control (Group 1), (**B**). Positive control (Group 2), (**C**). 5-FU@ZIF-8-SDG gel formulation (Group 8), (**D**). Marketed formulation (Group 9).

Visual observation was performed for 4 groups, namely, Group 1 (negative control; no disease-induced, no drug treatment), Group 2 (positive control; disease-induced no treatment), Group 8 (5-FU@ZIF-8-SDG complex dispersed 2% HPMC and 0.5% HA combination gel) and Group 9 (marketed formulation; 5% Efudex), as shown in Figure 7. When compared to Group 2, Group 8 and Group 9 visibly demonstrated a reduction in BCC after one month of treatment. Additionally, compared with the marketed formulation, the area of the skin where the 5-FU@ZIF-8-SDG MOF gel was applied showed a greater reduction in the tumor, indicating the improved pharmacodynamic profile of the drug in the presence of SDG.

(b) Histopathological evaluation

**Figure 8:**
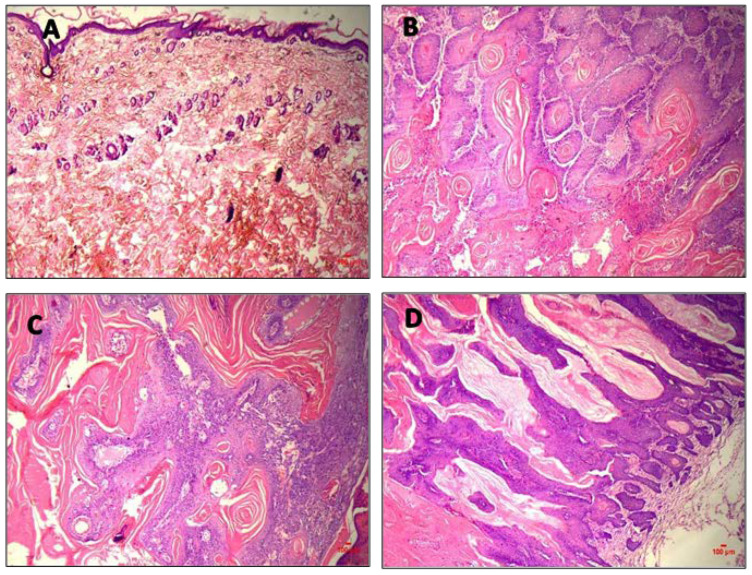
Photomicrographs of histopathological evaluation of rat skin stained with H and E Negative in in vivo pharmacodynamics study (Magnification: 40X). (**A**): Negative control (Group 1); (**B**): Positive control (Group 2); (**C**): Marketed topical cream (Group 9); (**D**): 5-FU@ZIF-8-SDG gel treated (Group 8).

The skin tissue from all the groups was excised, and adhering subcutaneous fat was scraped out, washed carefully with water, collected in 10% formalin solution and viewed under a microscope for structural changes that occurred during the treatment period. The slides stained with hematoxylin and eosin were checked for pathological changes in the treatment groups compared to the positive control under a light microscope. The parameters considered were keratinization, necrosis, inflammatory infiltration and nuclear pleomorphism. In the positive control group (Group 2), the epithelium was ulcerated with epithelial islands invading into the connective tissue. The epithelial cells showed well-established basilar hyperplasia, nuclear pleomorphism, acute and chronic inflammatory cell infiltration, keratin pearls, necrosis, and dyskeratosis, as shown in Figure 8. The ulcers observed in the epithelium and epithelial islands invading into the connective tissue were decreased in Groups 7, 8 and 9. The epithelial cells showed mild hyperplasia, nuclear pleomorphism, and dyskeratosis in many mitotic figures, indicating that the formulation effectively treats BCC.

(c) Immunohistochemistry

**Figure 9:**
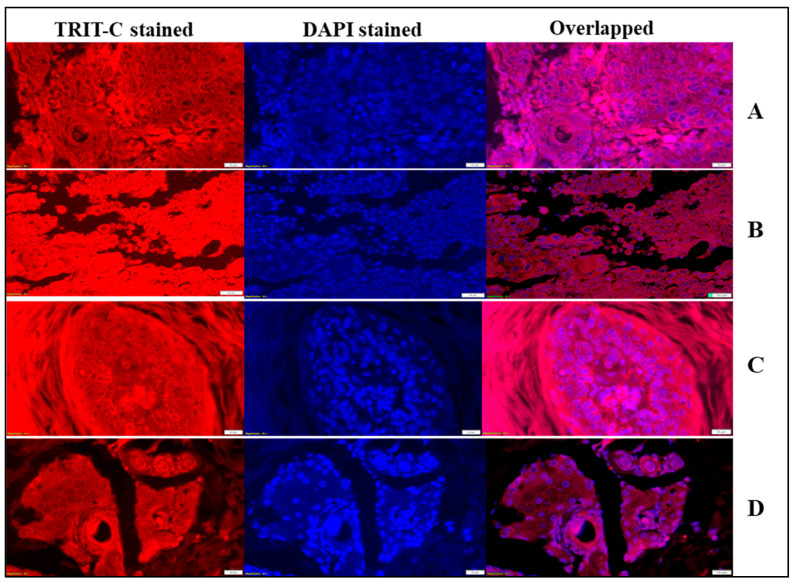
Immunohistochemistry results with different treatments. (**A**): Negative control; (**B**): Positive control; (**C**): 5-FU@ZIF-8-SDG-treated group; (**D**): Marketed cream.

The negative, low and high expression of Bcl-2 protein was observed under a fluorescence microscope, and an average of 10 images were taken from each treatment group for counting the cells (Figure 9). Scoring was performed based on Bcl-2 protein expression. Cytoplasmic immunoreactivity in the “Bcl-2 positive” subset of cancers ranged from 1 to 100% of the overall tumour tissue (mean value 70%). The expression of Bcl-2 protein was categorized as weak or strong based on the intensity of the staining. The Bcl-2 protein staining with mixed nodular-infiltrative appeared to be greater and more expressive than in deeper infiltrative neoplastic forms. When Bcl-2 protein status was statistically assessed in cancer tissue, i.e., low expression versus high expression, an association between 5-FU@ZIF-8-SDG MOF gel and other formulation-treated groups was confirmed. Bcl-2 protein expression was significantly lower in the 5-FU@ZIF-8-SDG gel-treated group than in the other treatment groups (*p* < 0.001). However, the positive control group showed good immunoreactivity with high expression of Bcl-2 protein, and the nonneoplastic epidermis of the hair follicles and Bcl-2 protein immunoreactivity were restricted to basal keratinocytes, whereas the upper and suprabasal layers were negative, as reported by Barto et al. [65]. The immunohistochemical findings in our set of BCCs are presented in Table 6.

(d) Protein expression study

**Figure 10:**
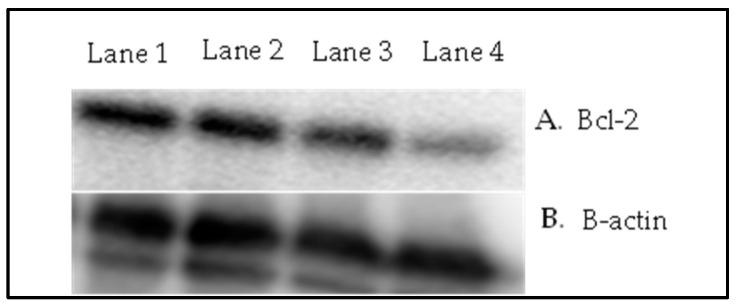
Results of Western blot analysis. (**A**) Representative protein expression analysis by Western blot of animal tissue fractions. Lane 1 is the disease control sample, which shows relatively higher expression of Bcl-2 protein than the treated groups (Lanes 2 to 4 are the plain 5-FU & SDG gel, marketed formulation and 5-FU@ZIF-8-SDG gel, respectively). (**B**) β-actin bands, which were used as an internal control.

Because there was no significant reduction in the BCC area in the other treatment groups, in the Western blot analysis, we focused only on three treatment groups, i.e., Group 5 (plain 5-FU & SDG gel), Group 8 (5-FU@ZIF-8-SDG gel) and Group 9 (marketed formulation), along with one disease control Group 2 (disease induced no treatment). The immunoblot analysis showed a higher intensity of Bcl-2 protein expression in Group 2 (Lane 1) than in Group 5, Group 8 and Group 9. Lanes 2 and 3 represent plain 5-FU & SDG gel and marketed formulation treated groups, respectively, showing the comparatively higher expression of Bcl-2 proteins. Lane 4, i.e., the 5-FU@ZIF-8-SDG gel-treated group, showed relatively low intensity Bcl-2 protein levels, confirming the effectiveness of the formulation. The results of western blot analysis are depicted in Figure 10.

### 3.9. Stability Studies

Based on the in vitro and in vivo evaluation studies, formulation 5-FU@ZIF-8-SDG gel and optimized dried sample of 5-FU@ZIF-8-SDG MOFs were selected for stability studies. The appearance, colour, odour, drug content, pH, viscosity and spreadability of the gel over six months are given in Table 7, and the stability of the optimized dried sample of 5-FU@ZIF-8-SDG is given in Table 8. The results showed that the formulation 5-FU@ZIF-8-SDG gel and 5-FU@ZIF-8-SDG MOFs were stable for six months under normal storage conditions.

## 4. Conclusions

In conclusion, an effective drug delivery system for the targeted treatment of skin cancer was established using multifunctional MOFs (5-FU@ZIF-8-SDG) in gel for topical administration. Using this approach, we were able to increase the 5-FU loading more efficiently and achieve complexation with SDG. Under physiological conditions, ex vivo permeation studies showed effective drug retention and moderate permeation in the skin, thereby improving its overall effectiveness. Confocal laser scanning microscopic studies indicated that the transfollicular pathway was the dominant route for skin permeation of the MOFs. The cytotoxicity assay and cell uptake studies proved the enhanced uptake of the optimized MOFs in the cancer cells with higher efficacy in destroying the cancer cells. The pharmacodynamics studies proved the enhanced efficacy of the optimized MOF gels based on visual, histopathological, immunohistochemistry and protein expression studies. Overall, this study offers a valuable tool for investigating the MOFs incorporated into gels as drug-targeted systems, and this strategy holds great potential for the administration of MOF gels in targeted topical treatment for BCC cancer.

## Figures and Tables

**Table 1:** Composition of different gel formulations.

Sl. No.	Ingredients	Plain Gel	Plain 5-FU Gel	Plain 5-FU& SDG Gel	ZIF-8 MOFs Gel	5-FU@ ZIF-8-SDG Gel
1	HPMC	200 mg	200 mg	200 mg	200 mg	200 mg
2	Hyaluronic acid	50 mg	50 mg	50 mg	50 mg	50 mg
3	Propylene glycol	200 µL	200 µL	200 µL	200 µL	200 µL
4	Methyl paraben	2 mg	2 mg	2 mg	2 mg	2 mg
5	Propyl paraben	1 mg	1 mg	1 mg	1 mg	1 mg
6	Triethanolamine	To adjust pH to 7	To adjust pH to 7	To adjust pH to 7	To adjust pH to 7	To adjust pH to 7
7	Water q.s.	10 mL	10 mL	10 mL	10 mL	10 mL
8	5-FU	---	500 mg	500 mg	---	500 mg
9	SDG	---	---	80 mg	---	80 mg
10	ZIF-8 MOFs	---	---	---	800 mg	800

--- = Not applicable.

**Table 2:** Physicochemical evaluation of prepared gel formulations.

Parameters	Plain 5-FU	Plain SDG	Plain 5-FU and SDG	ZIF-8 MOFs	5-FU@ZIF-8 MOFs	5-FU@ZIF-8-SDG
Appearance	Smooth	Smooth	Smooth	Smooth	Smooth	Smooth
Homogeneity	+++	+++	+++	+++	+++	+++
Color	Off-white	Off-white	Off-white	Off-white	Off-white	Off-white
Drug content (%)	98.15 ± 0.5	96.25 ± 0.15	97.12 ± 0.25 and 95.25 ± 0.15	NA	95.53 ± 0.25	95.12 ± 0.16 and 95.05 ± 0.18
pH	6.48 ± 0.02	6.43 ± 0.05	6.41 ± 0.08	6.45 ± 0.05	6.44 ± 0.04	6.4 ± 0.06
Spreadability (mm)	21.65 ± 0.21	22.62 ± 0.23	22.64 ± 0.11	22.59 ± 0.25	22.60 ± 0.18	22.63 ± 0.27
Viscosity (cp) at 50 rpm	33.6 ± 2.15	33.4 ± 1.45	34.5 ± 3.12	34.3 ± 3.05	33.2 ± 2.65	33.4 ± 3.52

+++: High; NA: Not applicable.

**Table 3:** Permeability parameters in skin permeation studies of 5-FU and SDG at pH 6.8.

Skin Permeation Studies	Q24 Values (µg/cm^2^)	Drug Content in Skin (µg/cm^2^)	Permeability Coefficient (cm/h)
Gel containing plain 5-FU and SDG	1569.22 ± 105.24 and 813.14 ± 294.85	998.16 ± 140.18 and 109.13 ± 10.12	0.0129 and 0.0118
5-FU@ZIF-8-SDG complex gel	2896.32 ± 187.50 and 1356.24 ± 90.72	3898.16 ± 120.18 and 615.26 ± 49.12	0.0186 and 0.0134

All the values are presented as Mean ± SD, n = 3; Q24 = Total amount of drug permeated at the end of 24 h.

**Table 4:** Primary skin irritation studies of gel formulation.

Formulation Treatment Groups	Reaction Grade		PII
	Erythema	Edema	
Control	0.00 ± 0.00 *	0.00 ± 0.00 *	0.00 ± 0.00 *
Positive control	2.33 ± 0.33	2.17 ± 0.31	2.25 ± 0.25
ZIF-8 MOFs gel	0.79 ± 0.05 *	0.64 ± 0.07 *	0.62 ± 0.06 *
Plain 5-FU and SDG gel	0.98 ± 0.05 *	0.67 ± 0.04 *	0.76 ± 0.05 *
5-FU@ZIF-8-SDG gel	0.84 ± 0.08 *	0.72 ± 0.02 *	0.74 ± 0.04 *

All values are expressed as Mean ± SD, n = 6. PII: primary irritation index. * Significantly (*p* < 0.05) different compared to the positive control. Erythema scale: 0 = none; 1 = slight; 2 = well defined; 3 = moderate; and 4 = scar formation; Edema scale: 0 = none; 1 = slight; 2 = well defined; 3 = moderate; and 4 = severe.

**Table 5:** Histopathological evaluation of rat skin.

Groups	Deg	Nec	Con	Inf	Ede
Control	---	---	---	---	---
Positive control	+	+	+	+++	+++
ZIF-8 MOFs gel	+	+	+	+	---
Plain 5-FU and SDG gel	+	+	+	+	+
5-FU@ZIF-8-SDG gel	+	+	+	+	---

Histopathological scale: --- = not observed; + = slight; ++ = moderate; +++ = severe; Deg = Degeneration; Nec = Necrosis; Con = Congestion; Inf = Inflammation; Ede = Edema.

**Table 6:** Immunohistochemical findings for negative, low, and high expression of Bcl-2 protein.

Treatment Group	Negative Bcl-2 Expression	Low Bcl-2 Expression	High Bcl-2 Expression
Group 1: Negative Control group (No disease indued)	0 (0%)	0 (0%)	0 (0%)
Group2: Control group (No treatment)	0 (0%)	15.5%	84.5%
Group8: 5-FU@ZIF-8-SDG MOF gel	5.4%	22.6%	72.3%

**Table 7:** Stability study of the optimized gel formulation (5-FU@ZIF-8-SDG gel) at 25 ± 2 °C and 60 ± 5% RH.

Parameters	Initial	1 Month	3 Months	6 Months
Appearance	Smooth, Homogeneous	Smooth, Homogeneous	Smooth, Homogeneous	Smooth, Homogeneous
Color	Off-white	Off-white	Off-white	Off-white
Drug content (%)	99.15 ± 0.16 and 99.24 ± 0.13	98.10 ± 0.10 and 98.93 ± 0.11	96.31 ± 0.18 and 97.86 ± 0.15	95.12 ± 0.16 and 96.05 ± 0.18
pH	6.48 ± 0.02	6.43 ± 0.05	6.41 ± 0.08	6.45 ± 0.05
Spreadability (mm)	22.62 ± 0.23	22.64 ± 0.11	22.59 ± 0.25	22.60 ± 0.18
Viscosity (cp)	34.5 ± 3.12	34.3 ± 3.05	33.6 ± 2.15	33.4 ± 1.45

All values are represented as Mean ± SD, n = 3.

**Table 8:** Stability study of the optimized formulation (5-FU@ZIF-8-SDG) in powder form at 25 ± 2 °C and 60 ± 5% RH.

Parameters	Initial	1 Month	3 Months	6 Months
Appearance	Crystalline	Crystalline	Crystalline	Crystalline
Color	Off-white	Off-white	Off-white	Off-white
Drug content (%)	99.15 ± 0.16 and 99.24 ± 0.13	99.10 ± 0.10 and 98.93 ± 0.11	98.31 ± 0.18 and 98.46 ± 0.15	98.12 ± 0.16 and 98.05 ± 0.18

All values are represented as Mean ± SD, n = 3.

## Data Availability

The datasets generated during the current study are available from the author on reasonable request.

## Supplementary Materials

The following supporting information can be downloaded at: https://www.mdpi.com/article/10.3390/pharmaceutics15112594/s1. Table S1. Composition of sodium citrate buffer (10 mM) solution pH 6.0; Table S2. Composition of SDS-PAGE gel; Table S3. Composition of Stripping buffer.
