# Supplementary material for: Targeted Delivery of 5-Fluorouracil and Sonidegib via Surface-Modified ZIF-8 MOFs for Effective Basal Cell Carcinoma Therapy
Source: Pharmaceutics. 2023 Nov 7;15(11):2594. doi: 10.3390/pharmaceutics15112594 (PMC10675485; doi:10.3390/pharmaceutics15112594)
Supplement: Supplementary file 1 [file pharmaceutics-15-02594-s001.zip › pharmaceutics-2619139-supplementary.pdf]

Supplementary Information

Targeted Delivery of 5-Fluorouracil and Sonidegib via Surface-Modified ZIF-8 MOFs for Effective Basal Cell Carcinoma Therapy

Bharath Singh Padya, Gasper Fernandes, Sumukha Hegde, Sanjay Kulkarni, Abhijeet Pandey, Sheikh F. Ahmad, Praful Balavant Deshpande4 Dinesh Upadhya, Srinivas Mutalik*

S1 Methods

S1.1. Immunohistochemistry

The tissues, collected for histopathological studies, were also used for immunohistochemistry study to identify the suppression of Bcl-2 gene expression in the treated group by fluorescent microscope. The detailed procedure is given below. Initially buffer was prepared for heat-induced epitope retrieval method. The formula for this buffer is given below:

Table S1. Composition of sodium citrate buffer (10 mM) solution pH 6.0

**S. No**	**Ingredients**	**Quantity**
1	Tri-sodium citrate (dihydrate)	2.94 gm
2	Tween 20	0.5 mL
3	Milli-Q water	1000 mL
4	Hydrochloric acid (1N)	To adjust pH

The above-prepared buffer solution was stored at 4 °C. The sections were subjected for Heat induced antigen (epitope) retrieval method using a water bath.

- To deparaffinize and rehydrate the sections, a clean beaker added with sufficient (around 500 mL) antigen retrieval solution (sodium citrate buffer solution pH 6.0) was taken and kept in a water bath.
- The temperature of the water bath was set at 60 °C, and 4 slides were placed in the beaker and kept overnight. On the next day, the slides were taken out from the beaker carefully and washed with 1x PBS before using it for immunostaining.

Steps involved in Immunostaining procedure for antigen retrieved histology slides:

1. The slides were washed gently with 500 µL of 1x PBS (filtered).
2. The sides of the slides was marked with a pap pen and allow the solution to dry

3. For fixation, 500 µL of 4% cold paraformaldehyde (PFA) in water (pH 7.4) was added to the well/ marked area and incubated it for 20 min at room temperature.
4. After incubation time, the PFA solution was removed, and the slides were washed with 500 µL of 1x PBS for three times, 3 min each.
5. For Permeabilization & Blocking, 2 µL Triton X 100 (TX100) and 50 µL of Donkey serum was added to 450 µL 1x PBS. This solution was added to the slides and incubated at room temperature for 30 min.
6. Staining:
    - Working solution consisted of: 475 µL of 1x PBS + 2 µL TX100 + 25 µL Donkey serum + 1 µL of Bcl-2 primary antibody.
    - Added 500 µL of above antibody solution to the slide and incubated overnight (14-16 h) at 4 ^{o}C in a dark condition.
    - On the next day, the antibody solution was removed by micropipette, and the slides were washed with 1x PBS for 5 times, 5 min each.
    - Added a secondary antibody solution (475 µL 1x PBS + 2 µL TX100 + 25 µL Donkey serum + 1µL of Alexa flour 568 anti-rabbit antibody depending on dilutions) on the slides and incubated at room temperature for 1 h in dark condition.
    - The secondary antibody solution was removed, and the slides were washed with 1x PBS for 5 times, 5 min each.
7. Mounting:
    - DAPI (1 µL in 300 µL 1x PBS) was added on each slide and incubated at room temperature for 10 min. The slides were washed with 1x PBS for 3 times, 5 min each.
    - Added 10 µL of Antifade solution in PBS to the glass slide and placed a coverslip over the slide.
    - The slides were then observed under the fluorescent microscope for expression of protein.

S1.2 Protein expression study

Western blot analysis was performed in the collected skin tissue to quantify the amount of Bcl-2 protein expressed in different treatment groups. The steps involved in the protein expression study are as follows:

a) Sample preparation using tissue lysate

The skin tissue was taken in an Eppendorf tube and placed in the ice. Then tissue lysate solution (RIPA lysis buffer; ≈ 200 µL) was added into the tube and homogenate using a hand homogenizer. After homogenization, 400 µL of RIPA buffer solution + 4 µL of protease inhibitor cocktail buffer was added and kept for 30 min in ice. Homogenate was sonicated in Probe sonicator (PRO650, Labman, Chennai, India) for 15 sec at 30 Hz and 4 cycles (each cycle consisted of 4 sec "On" and 2 sec "Off") to complete the tissue lysis. The homogenate was centrifuged at 14000 rpm for 20 min in a 4 °C precooled centrifuge. After centrifugation, the supernatant was removed gently and transferred to a fresh tube and stored it at -80 °C for Western blot analysis. The sample was thawed and, from the total volume, 25 µL of lysate was taken to perform the protein assay using 200 µL BCA reagents to determine the protein concentration at 595 nm. For Western blot analysis, an appropriate amount of protein sample was taken based on the protein assay value, and to that, an equal volume of 4x Laemmle buffer was added. The sample was heated at 95 °C for 5 min before the Western blot experiment.

b) Preparation of Sodium dodecyl sulphate poly acrylamide gel electrophoresis SDS-PAGE

For the preparation of 10% SDS-PAGE gel, the ingredients were mixed in the following order:

Table S2. Composition of SDS-PAGE gel

S. No	Ingredients	Quantity
1	Milli-Q Water	4.1 mL
2	Acrylamide/ bis (30% 37.5:1; Bio-Rad)	3.3 mL
3	Tris-HCl (1.5M, pH 8.8)	2.5 mL
4	SDS, 10%	100 µL
5	N,N,N,N-tetramethylethylene-diamine (TEMED)	10 µL
6	Ammonium persulfate (APS) 10%	32 µL

NOTE: TEMED and APS were added only when the contents are ready to pour because by adding these two, the "Separation Gel" will polymerize quickly and get solidified.

c) Protein separation by gel electrophoresis:

Prepared 10% gel was poured manually and allowed to solidify by placing a 10-well comb on it to load equal amounts of protein into the wells along with a BioRad pre-stained dual marker.

The gel was run for 5 min at 50 V and the voltage was increased to 100 V to finish the run in about 1 h.

d) Transferring the protein from the gel to the membrane:

Placed the gel in 1x Transfer Buffer for 10-15 min. Assembled the gel on the Sandwich containing nitrocellulose membrane, and no air bubble is trapped between the Sandwich and the membrane. The whole cassette was kept in the transfer tank and an ice block was placed in the tank. The Transferring current was set at 20 V for 2.5 h.

e) Antibody incubation:

After the transfer process, the blot was rinsed using Tris-Buffered Saline, 0.1% Tween® 20 Detergent (TBST), and stained with Ponceau S solution to check the quality of protein transfer. Washed the stain with TBST thrice for 5 min each and blocked the membrane with 5% non-fat milk for 1.5 h. Incubated the blot with primary antibody Bcl-2 (1:1000 dilution) overnight at 4 °C with a constant shaker. On the next day, the blot was washed three times with TBST for 10 min each and incubated the blot with anti-rabbit secondary antibody (1:5000) for 1 h at room temperature. Again, washed the blot three times with TBST for 5 min each.

f) Mild Stripping Protocol:

Mild stripping buffer was prepared as per the composition given below. All the ingredients were dissolved in Milli-Q water and the pH of the solution was checked for 2.2, and the volume was made to 1000 mL.

Table S3 Composition of Stripping buffer

**S. No**	**Ingredients**	**Quantity**
1	Glycine	15 gm
2	SDS	1 gm
3	Tween 20	10 mL
4	Milli-Q water	Up to 1000 mL

Added 10 ml of mild stripping buffer in a small plastic container and incubated the blot at room temperature for 10 min. The buffer was discarded, and the procedure was repeated with a fresh stripping buffer. Discarded the stripping buffer and add fresh 1x PBS buffer, and incubate the blot for 10 min. After 10 min, discarded the buffer and repeated the procedure with the fresh 1x PBS buffer. The blot was washed for 5 min with 1x TBST and repeated the procedure twice.

All the washing steps were carried out in the 2D shaker with constant shaking. Now the blot is ready for the blocking.

g) Steps for B- Actin for the same blot:

Blocked the membrane with 5% non-fat milk for 1.5 h. The blot was incubated with primary antibody B-actin (1:5000 dilution) overnight at 4 °C with a constant shaker. On the next day, the blot was washed three times with TBST for 10 min each and incubated the blot with anti-mouse secondary antibody (1:10000) for 1 h at room temperature. Again, washed the blot three times with TBST for 5 min each.

h) Imaging and data analysis:

Applied the chemiluminescent substrate to the blot according to the manufacturer's instructions. Chemiluminescent signals were captured using a CCD camera-based imager and the image was analysed using the Image J software.
